# Can switching from cigarettes to heated tobacco products reduce consequences of pulmonary infection?

**DOI:** 10.1186/s12931-024-02992-y

**Published:** 2024-10-19

**Authors:** Tariq A. Bhat, Suresh G. Kalathil, Noel J. Leigh, Maciej L. Goniewicz, Yasmin M. Thanavala

**Affiliations:** 1 Department of Immunology, Roswell Park Comprehensive Cancer Center, 665 Elm Street, Buffalo, NY 14263 USA; 2 Department of Health Behavior, Roswell Park Comprehensive Cancer Center, Buffalo, NY USA

**Keywords:** Tobacco product switching, Smoking cessation, IQOS, Heated tobacco products (HTPs), Combustible cigarettes, Respiratory infection, NTHI

## Abstract

**Rationale:**

While tobacco industry data suggests that switching from combustible cigarettes to heated tobacco products (HTPs), like IQOS, may reduce the users’ exposure to respiratory toxicants, it is not known if using HTPs impacts the outcomes of acute respiratory infections.

**Objectives:**

Does switching from cigarettes to HTPs improve responses to pulmonary infection.

**Methods:**

We conducted experiments in which 3 groups of mice were pre-exposed to cigarette smoke for 8 weeks, followed by 8-week exposure to (1) HTPs (*tobacco product switching*), (2) air (*smoking cessation*), or (3) continued exposure to cigarette smoke. Pulmonary bacterial clearance and surrogate markers of lung damage were assessed as study outcomes.

**Main results:**

Significantly compromised clearance of bacteria from the lungs post-acute challenge occurred in both the *switching* group and in mice continuously exposed to cigarette smoke. Bacterial clearance, inflammatory T-cell infiltration into the lungs, and albumin leak improved at 12 h post-acute challenge in the *switching* group compared to mice continuously exposed to cigarette smoke. Bacterial clearance, total lung immune-cell infiltration, inflammatory T-cell infiltration into the lungs, the content of total proteins in the BAL, and albumin leak measured post-acute challenge were compromised in the *switching* group compared to mice in the *cessation* group. Switching from cigarettes to HTPs did not improve lung myeloperoxidase and neutrophil elastase levels (markers for lung inflammation and damage), which, however, were significantly reduced in the *cessation* group.

**Conclusions:**

This study reveals only a modest improvement in respiratory infection outcomes after switching exposure from cigarettes to HTPs and significantly compromised outcomes compared to a complete cessation of exposure to all tobacco products.

**Supplementary Information:**

The online version contains supplementary material available at 10.1186/s12931-024-02992-y.

## Background

While there are many forms of tobacco products, the smoking of combustible cigarettes is the most common form of tobacco consumption worldwide. Smoking cigarettes is a significant risk factor for the development of numerous diseases, including cancers, respiratory, cardiovascular, and oral diseases [1–4]. Inhalation of cigarette smoke causes pulmonary inflammation and detrimental changes in the airway epithelium, resulting in lung damage [4–9]. It has also been well-documented that smoking cigarettes suppresses and slows down pulmonary clearance of acute bacterial infections and exacerbates infection-induced lung inflammation and damage, enhancing susceptibility to respiratory infections [4–9]. Evidence from the literature clearly supports smoking cessation as an efficient way to reduce the detrimental effects of cigarette smoke on pulmonary function and immunity to respiratory infections [4, 9].

Although e-cigarettes are the most popular alternatives to combustible cigarettes, all major tobacco companies have recently introduced heated tobacco products (HTPs), also called heat-not-burn (HnB) products, to the global market. HTPs and e-cigarettes are different in the way that they deliver nicotine. E-cigarettes heat a nicotine solution while HTPs heat the tobacco. Since tobacco is not burnt in HTPs, the levels of emitted harmful and potentially harmful chemicals (including many respiratory toxicants) are significantly reduced compared to levels found in cigarette smoke [10]. According to industry claims, HTPs were designed as potential harm-reducing products for adult smokers who would otherwise continue to smoke cigarettes [11]. Philip Morris International (PMI), the largest manufacturer of cigarettes, launched its HTP in 2016 under the IQOS brand name. IQOS is available in 73 markets worldwide [12]. In the US, IQOS was authorized for marketing as a modified risk tobacco product (MRTP) by the U.S. Food and Drug Administration (USFDA) on July 7, 2020 [13] and is expected to be available on the US market in mid-2024 [14]. According to PMI estimates, as of February 9, 2023, 17.8 million adult smokers globally have already switched to its IQOS HTP and completely stopped smoking cigarettes [12].

Most of the data on the health effects of HTPs come from industry-sponsored research that focuses on the potential benefits of switching from combustible cigarettes to HTPs. PMI data from in vitro study suggest that exposure of human organotypic epithelium cultures (buccal, bronchial, and nasal) to emissions from IQOS reduces the induction of inflammatory and cell stress responses compared to cigarette smoke [15]. PMI also reported in vivo data from an animal exposure model, showing that switching exposure from combustible cigarettes to HTP considerably impeded the progression of cigarette smoke-induced emphysematous and atherosclerotic changes in mice [16]. Human data provided by PMI showed that individuals who switched from combustible cigarettes to IQOS significantly reduced exposure to harmful and potentially harmful constituents to levels approaching those detected after smoking cessation. Furthermore, the effect of exposure reduction was associated with overall improvements in biomarkers of inflammation and oxidative stress [17, 18]. While switching to HTPs like IQOS appears to represent a better option for current smokers who would otherwise continue smoking, it does not automatically mean these products are risk-free. We recently showed that HTP aerosols induce pulmonary toxicity comparable to cigarette smoke and reduce the efficacy of vaccination against respiratory infection in mice [19]. In fact, independent evaluation of PMI data showed that IQOS aerosol exposures induce pulmonary inflammation and can potentially suppress pulmonary immunity and impair lung function [20, 21]. We also reviewed the industry-supported studies and identified an important knowledge gap about the potential effects of HTPs on respiratory infections.

While epidemiological studies are currently not available to elucidate the long-term health effects of HTP use, chronic inhalation exposure animal models can provide a valuable understanding of the effects of HTPs on the lungs. We have previously established an animal model of exposure to emissions from combustible cigarettes and alternative tobacco products, including e-cigarettes and HTPs [22]. Using this model, we designed the current study to assess whether switching from cigarettes to HTP can impact the outcome of an acute respiratory infection. Importantly, we also included a comparison of the potential benefits of switching from cigarettes to HTP to the ultimate cessation strategy of all tobacco products. The primary aim of our study was to evaluate whether switching from cigarettes to HTP reduces detrimental effects on responses against an acute pulmonary bacterial infection compared to continued exposure to cigarette smoke. The secondary aim was to compare responses after switching from cigarettes to HTP with responses observed after cessation of tobacco products.

## Materials and methods

### Overview of the study protocol

**Fig. 1:**
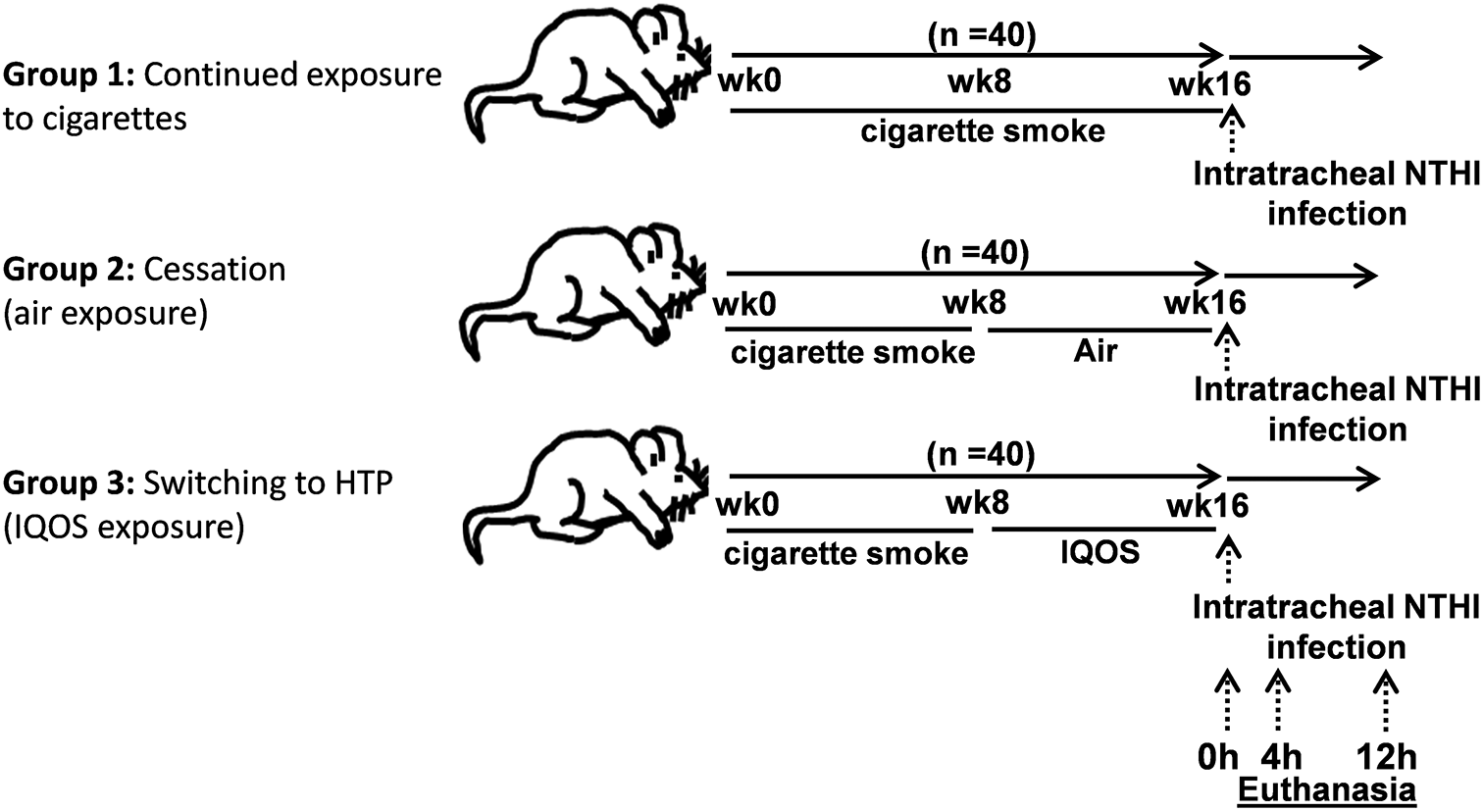
Schema of exposures. The figure depicts the schema of exposures in animals to various aerosols in the study

C57BL/6NCr mice (*n* = 120; 60 male and 60 female) were pre-exposed for 8 weeks to cigarette smoke. After 8 weeks, mice were randomly assigned to three different exposure conditions for the next 8 weeks: (1) *continued exposure* to cigarette smoke, (2) *cessation* group (air exposure), or (3) product *switching* group (HTP), (*n* = 40 mice/group; 20 male and 20 female; Fig. 1). After 16 weeks, all animals were challenged by intratracheal instillation of nontypeable *Haemophilus influenzae* (NTHI), an opportunistic gram-negative bacterium responsible for respiratory tract infections and exacerbations in COPD patients [23], and euthanized 0, 4, and 12 h later. Animal procedures were approved by the Institutional Animal Care and Use Committee and complied with state, federal, and NIH regulations.

### Animal exposure conditions

All exposures were done for 5 h/day, 5 days/week, for 16 weeks. Eight-week old mice were exposed to an equivalent dose of nicotine delivered from HTPs and combustible cigarettes. Nicotine equivalency was determined by quantifying serum cotinine levels (a nicotine metabolite) in blood samples collected 30 min after exposure. Despite differences in the puffing protocols used, we achieved equivalent exposure to nicotine at weeks 9–16 (Table 1). Details of exposure conditions and analytical methods used to monitor exposure are provided in the Supplementary Materials.

**Table 1:** Exposure conditions to emissions from HTPs, combustible cigarettes, and air (*control*)

	Weeks 1–8	Weeks 9–16		
	Cigarettes	HTP	Cigarettes	Cessation (Air)
Puffing protocol				
Number of products used over 5 h	60 cigarettes	20 tobacco sticks	60 cigarettes	**--**
Number of puff clusters over 5 h	60	20	60	**--**
Number of puffs per cluster	8	12	8	**--**
Total number of puffs taken over 5 h	480	240	480	**--**
Interval between puff clusters	1 min	9 min	1 min	**--**
**Airborne exposure**				
Airborne Nicotine (µg/m^3^)	581.3 ± 420.1	196.3 ± 144.1	549.2 ± 301.4	17.7 ± 13.6
PM_5.0_ (mg/m^3^)	10.5 ± 8.9	1.5 ± 7.2*	11.3 ± 10.7*	< LOQ
TPM (mg/m^3^)	182.8 ± 69.6	35.1 ± 13.5*	198.4 ± 73.1*	< LOQ
**Thirdhand exposure**				
Nicotine deposited on surface (mg/m^2^/5 h)	0.3 ± 0.3	0.4 ± 0.4	0.2 ± 0.1	< LOQ
Nicotine deposited on fur (mg/m^2^/16 wks)	3.2 ± 0.7	4.8 ± 1.9	2.8 ± 0.8	< LOQ
**Systemic nicotine exposure**				
Serum cotinine in females (ng/mL)-	39.5 ± 22.5	26.4 ± 13.6	24.0 ± 9.3	< LOQ
Serum cotinine in males (ng/mL)	24.9 ± 17.8	22.3 ± 10.9	25.5 ± 11.5	< LOQ
Serum cotinine in female + male (ng/mL)	31.4 ± 21.3	25.7 ± 13.2	24.4 ± 10.6	< LOQ

Note: Limits of quantitation (LOQ) were as follows: airborne nicotine 0.3 µg/m^3^, nicotine deposited on surface 0.1 mg/m^2^, nicotine deposited on fur 2.2 mg/m^2^, serum cotinine 5.0 ng/ml. *A significant difference in aerosol concentration between HTP and cigarette exposure conditions at weeks 9–16 (*p* ≤ 0.05)

### Assays

After 16 weeks of cumulative exposure (and before the infection challenge), half of the animals were given intratracheal FITC-dextran to measure the plasma levels of FITC-dextran one hour later as an index of lung-endothelial damage [19, 24]. Two days later, all animals were challenged by intratracheal instillation of NTHI bacteria and euthanized at 0, 4, and 12 h after challenge as per the *schema of exposures* depicted in Fig. 1. Upon euthanasia, BAL and lungs were harvested to measure the post-infection outcomes assessed at 0, 4, and 12 h after bacterial challenge. These included:

- bacterial clearance from lungs;
- pulmonary inflammatory microenvironment evaluated by quantifying infiltration of immune cells in lung tissue;
- markers of lung-epithelial and -endothelial damage, including systemic FITC-dextran leak from lungs, total BAL protein accumulation and albumin leak in the BAL; and.
- markers of tissue remodeling during the inflammatory response, including myeloperoxidase (MPO) and neutrophil elastase (NE) levels in lung tissue.

Details about each assay used in the study are provided in Supplementary Materials.

### Statistical analysis

Statistical analyses were like those described previously [24]. Statistically significant differences between the mean rank values of different exposure groups (continued exposure to cigarette smoke, switching, and cessation) were determined by performing Kruskal-Wallis’s non-parametric test. *P* values were corrected for multiple testing using the ‘two-stage linear step-up procedure of Benjamini, Krieger, and Yekutieli’ false discovery rate (FDR) method, and the differences between the two groups were considered statistically significant at *p* < 0.05 when FDR was set at Q < 0.1. Using the same statistical approach, we also examined differences between male and female mice in their responses to different exposure conditions. All statistical analyses were done using GraphPad Prism 9.5.1 software (GraphPad; La Jolla, California, USA). Data are shown in figures as mean ± SE.

## Results

### Pre-infection assessment: switching to HTP marginally reduces cigarette smoke-induced lung endothelial damage

**Fig. 2:**
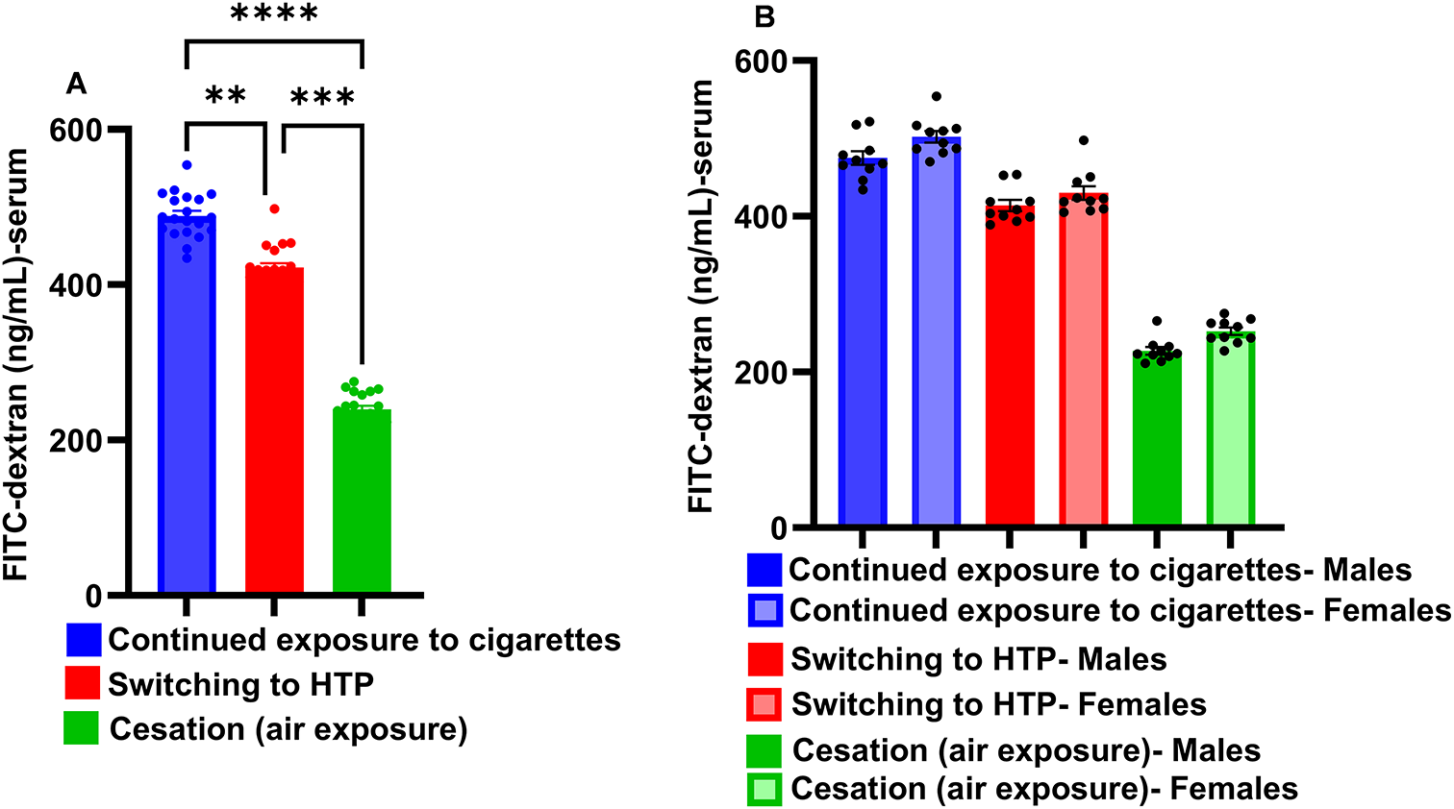
Lung-endothelial damage induced at the end of inhalation exposure. At the end of exposures, intratracheal instillations of FITC-dextran were given to mice and the levels of FITC-dextran leaking into plasma were quantified one hour later to measure the extent of lung-endothelial damage as described in detail in methods. **Panel A** represents data from males + females combined, while **panel B** represents data as males versus females. Difference between two groups is considered significant at *p* < 0.05 and statistical significance of the difference between two groups is indicated with symbols **p < 0.01; ***p < 0.001; ****p < 0.0001 after performing non-parametric Kruskal-Wallis test with false discovery rate (FDR) correction for multiple comparisons by GraphPad Prism V.9 software (GraphPad; La Jolla, CA, USA). In each experiment 10 males and 10 females per exposure group were used. Results are depicted as mean ± SE

Switching exposures from combustible cigarettes to IQOS resulted in less lung endothelial damage, as manifested by a modest but significant decrease in the plasma levels of FITC-dextran, leaked into the circulatory system from bronchoalveolar space, compared to continued exposure to cigarette smoke (*p* < 0.01) (Fig. 2A). However, complete cessation of cigarette smoke exposure very markedly reduced lung endothelial damage compared to continued exposure to cigarette smoke (*p* < 0.0001), and this strategy was much better at protecting mice from lung damage compared with switching to IQOS (*p* < 0.001). No differences in lung endothelial damage were observed between males and females following these exposures (Fig. 2B).

### Switching to HTP induces a modest enhancement in cigarette smoke-suppressed bacterial clearance from the lungs

**Fig. 3:**
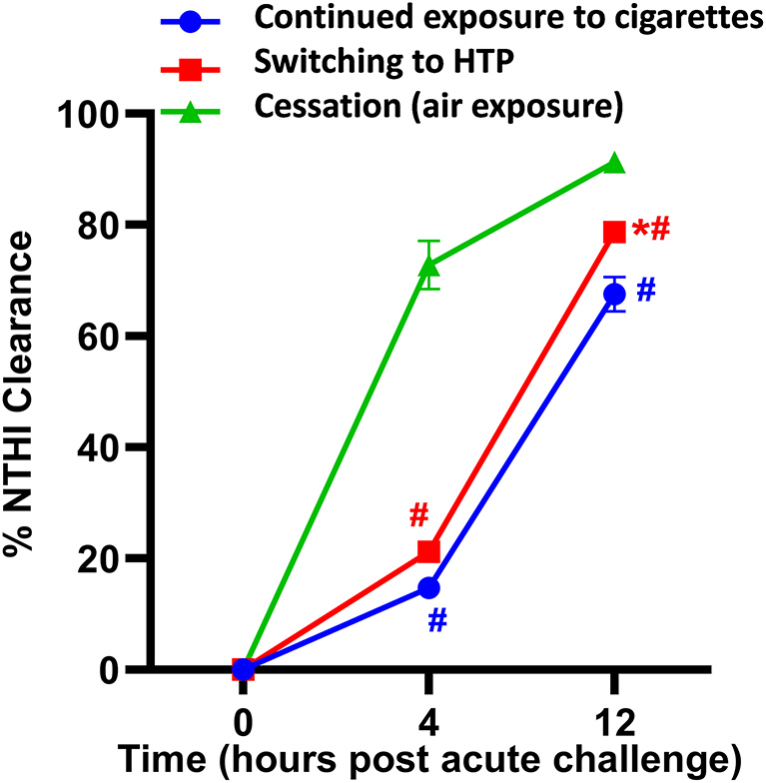
NTHI clearance from the lungs. Two days after exposures ended, all animals were challenged by intratracheal instillation of 1 million colony forming units of NTHI and euthanized at 0, 4, and 12 h after challenge. Lungs were harvested, tissue homogenates prepared, and various dilutions of the homogenate plated onto chocolate agar plates to grow and count NTHI colony forming units as described in detail in the supplemental methods. Data are presented as %NTHI clearance as a measure of lung bacterial burden. Difference between two groups was considered significant at *p* < 0.05 using Kruskal-Wallis test corrected for multiple comparisons by controlling the False Discovery Rate using Two-stage linear step-up procedure of Benjamini, Krieger and Yekuieli post-test by GraphPad Prism V.9 software (GraphPad; La Jolla, California, USA). Desired FDR was adjusted at Q = 0.1. * Statistically significant difference from continued exposure to cigarettes (blue). # Statistically significant difference from cessation (green). Numbers of mice used for: continued exposure to CS group (*n* = 8 mice at 0, *n* = 8 mice at 4 h and *n* = 6 mice at 12 h timepoint); for switching to HTP group (*n* = 12 mice at each 0, 4 and 12 h timepoints), and for cessation group (*n* = 10 mice at 0 h, *n* = 8 mice at 4 h and *n* = 6 mice at 12 h timepoint). Results are depicted as mean ± SE

While there was no difference in the clearance of NTHI bacteria 4 h post-acute challenge in the switching group compared to the continued exposure group, the bacterial clearance at 12 h was significantly greater in the switching group (Fig. 3). However, bacterial clearance in a cessation group was markedly enhanced at 4 h and 12 h time points post-acute challenge compared to switching and continued exposure groups (Fig. 3).

### Switching from combustible cigarettes to HTP modulates immune-cell infiltration into the lungs following acute bacterial challenge

**Fig. 4:**
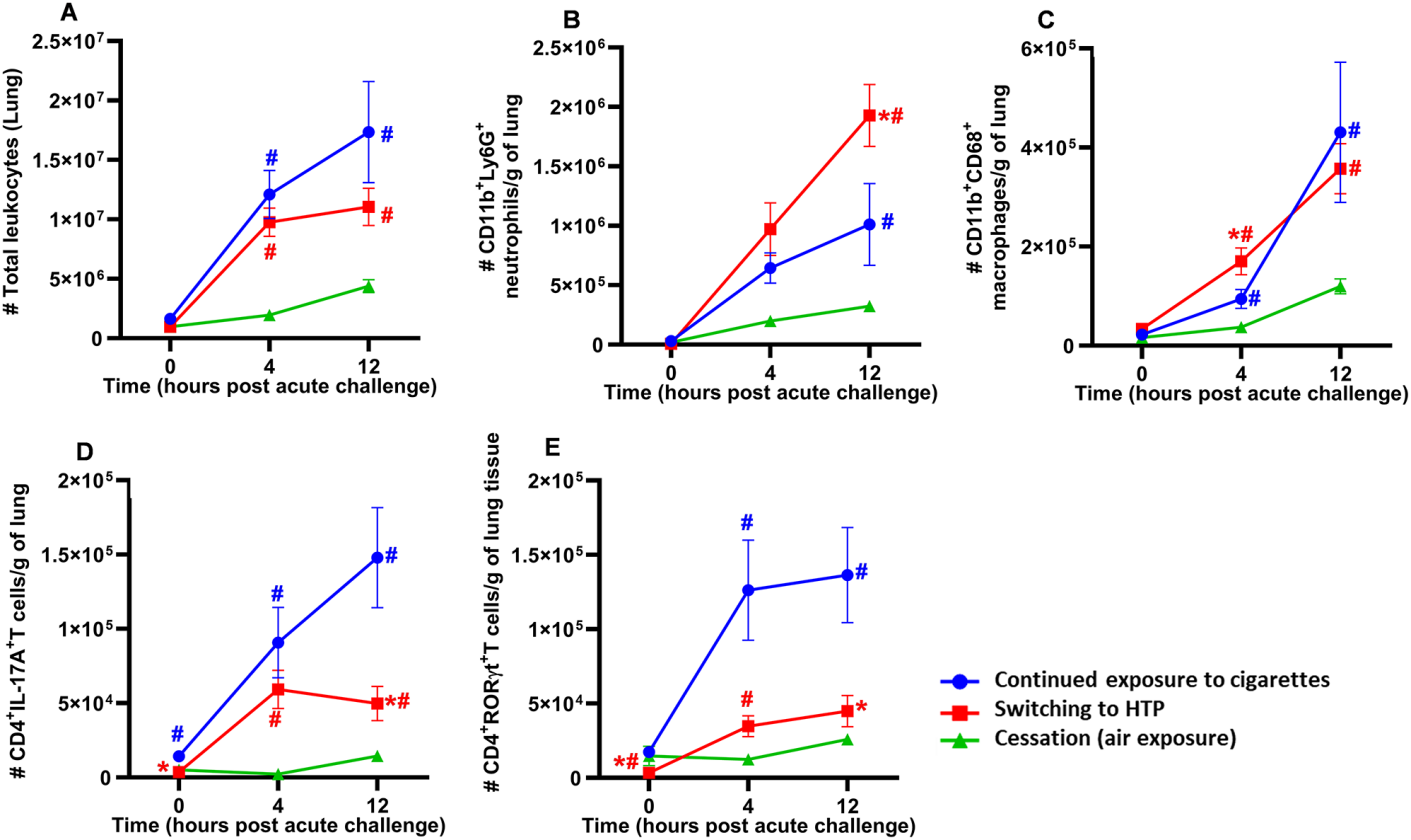
Impact of exposures on pulmonary immune-cell infiltration. Total number of leucocytes (**A**), CD11b^+^Ly6G^+^ neutrophils (**B**), CD11b^+^CD68^+^ macrophages (**C**), CD4^+^IL17A^+^ T-cells (**D**) and CD4^+^RORγt^+^ inflammatory T-cells (**E**) in the lungs of exposed-mice infected with NTHI for 0, 4, and 12 h were determined by flow cytometry using specific markers and following a gating strategy as described previously (9, 22, 19) and shown in supplemental figure E1. Difference between two groups is considered significant at *p* < 0.05 using the Kruskal-Wallis test corrected for multiple comparisons by controlling the False Discovery Rate using Two-stage linear step-up procedure of Benjamini, Krieger and Yekuieli post-test. Desired FDR was adjusted at Q = 0.1. * Statistically significant difference from continued exposure to cigarettes (blue). # Statistically significant difference from cessation (green). * Statistically significant difference from continued exposure to cigarettes (blue). # Statistically significant difference from cessation (green). Numbers of mice used for: continued exposure to CS group (*n* = 8 mice at 0, *n* = 11 mice at 4 h and *n* = 8 mice at 12 h timepoint); for switching to HTP group (*n* = 12 mice at each 0, 4 and 12 h timepoints), and for cessation group (*n* = 10 mice at 0 h, *n* = 12 mice at 4 h and *n* = 8 mice at 12 h timepoint). Results are depicted as mean ± SE

We found that compared to the continued cigarette smoke exposure group, mice that switched to IQOS exposure showed no significant decrease in the total numbers of leukocytes in the lung tissue at 0 h, 4–12 h after acute bacterial challenge (Fig. 4A). However, the cessation group showed significantly reduced infiltration of leukocytes into the lung tissue at all time points (0, 4, and 12 h after acute bacterial challenge) compared to the group continuously exposed to cigarette smoke (Fig. 4A). Additionally, the infiltration of leukocytes in the lungs of mice in the cigarette cessation group significantly decreased when compared to mice that had been switched to IQOS (Fig. 4A).

Switching exposure from cigarettes to IQOS did not change lung neutrophil numbers at 0 h and 4 h post-acute challenge compared to continued exposure to cigarette smoke. However, their numbers were significantly augmented in the switch group at 12 h post-acute challenge compared to continued exposure to cigarette smoke (Fig. 4B). Cessation of exposure to cigarette smoke resulted in a significant reduction in the numbers of neutrophils at 12 h after acute bacterial challenge compared to the switching group but not to the continued exposure group (Fig. 4B). Switching exposure from cigarettes to IQOS did not impact the numbers of CD68^+^ macrophages at 0 and 12 h following acute bacterial challenge but augmented these cells after 4 h (Fig. 4C). However, macrophage numbers in the cessation group were significantly reduced at 4 h and 12 h after acute bacterial challenge compared to the switching and continued exposure groups (Fig. 4C).

Switching cigarette exposure to IQOS significantly diminished proinflammatory CD4 + IL-17 A + T-cell infiltration into the lungs at 0 h and 12 h after acute bacterial challenge compared to continued exposure to cigarette smoke (Fig. 4D). However, cessation of exposure resulted in a marked reduction in the infiltration of these inflammatory T-cells at all time points after acute bacterial challenge. Compared to continued exposure to cigarette smoke, switching exposure from cigarettes to IQOS significantly reduced the numbers of CD4^+^RORgt^+^ T-cells in the lungs at 0 h and 12 h after acute bacterial challenge (Fig. 4E). Cessation of cigarette smoke exposure drastically diminished CD4^+^RORgt^+^ T-cells in the lungs at 0 h, 4 h and 12 h when compared to continued exposures groups, and at 0 h and 4 h when compared to switching (Fig. 4E). No difference in the modulation of pulmonary immune-cell infiltration was observed for any cell type between males and females following any of these exposures following acute bacterial infection (Supplemental Figure E2A-E).

### Switching from cigarettes to HTP does not reduce bacterial-induced lung epithelial damage

**Fig. 5:**
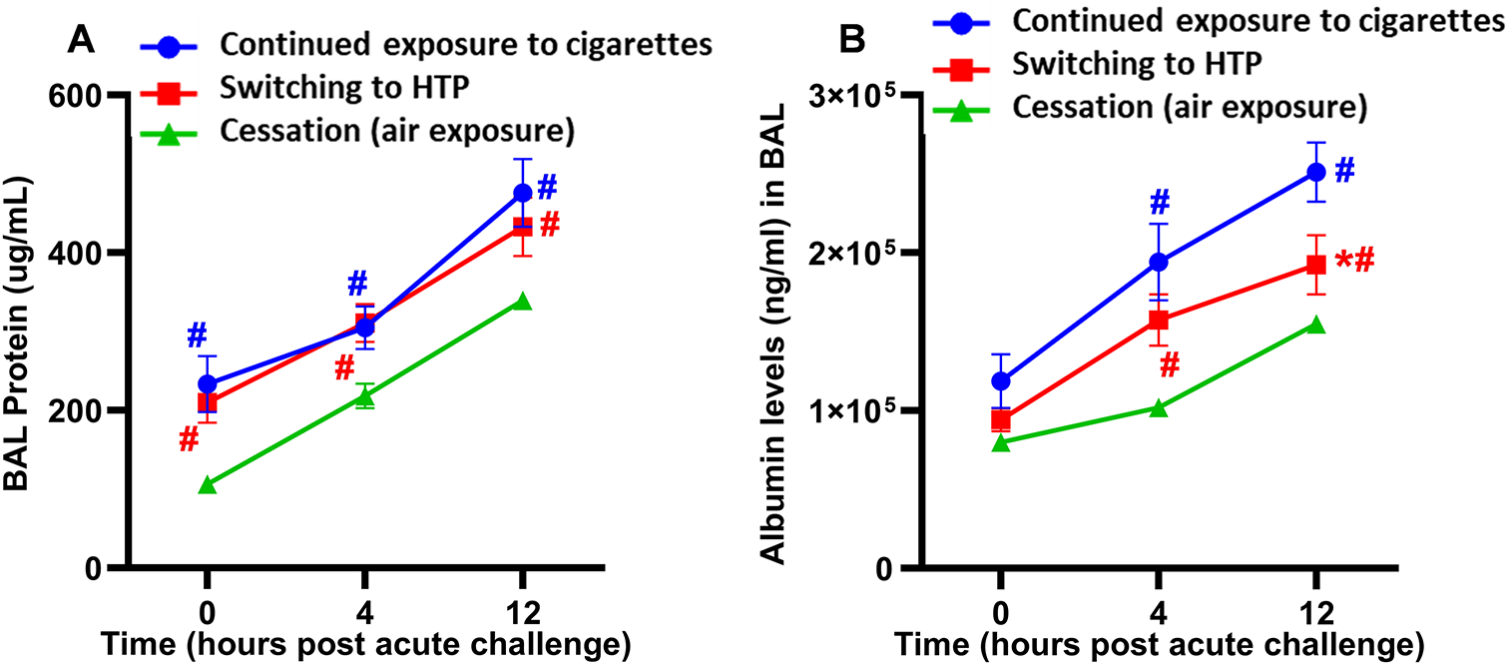
Markers of lung epithelial damage induced after inhalation exposure were quantified. Levels of (**A**) total proteins and (**B**) albumin in the BAL of exposed-mice infected with NTHI for 0, 4, and 12 h were quantified as described in the Materials and Methods section. Difference between groups is considered significant at *p* < 0.05 using the Kruskal-Wallis test corrected for multiple comparisons by controlling the False Discovery Rate using Two-stage linear step-up procedure of Benjamini, Krieger and Yekuieli post-test. Desired FDR was adjusted at Q = 0.1. * Statistically significant difference from continued exposure to cigarettes (blue). # Statistically significant difference from cessation (green). * Statistically significant difference from continued exposure to cigarettes (blue). # Statistically significant difference from cessation (green). Numbers of mice used for: continued exposure to CS group (*n* = 8 mice at 0, *n* = 11 mice at 4 h and *n* = 8 mice at 12 h timepoint); for switching to HTP group (*n* = 12 mice at each 0, 4 and 12 h timepoints), and for cessation group (*n* = 10 mice at 0 h, *n* = 12 mice at 4 h and *n* = 8 mice at 12 h timepoint). Results are depicted as mean ± SE

Levels of total proteins in the BAL following switching to HTP were equivalent at all time points after acute bacterial challenge compared to continued exposure (Fig. 5A). However, the cessation group showed significantly lower levels of total BAL proteins at all time points compared to the switching and continued exposure groups (Fig. 5A). Levels of albumin in the BAL did not differ at 0 h and 4 h after acute bacterial challenge in the switching group compared to the continued exposure group (Fig. 5B). However, albumin levels in the BAL were significantly decreased at 12 h after acute bacterial challenge in the switching group compared to the continued exposure group (Fig. 5B). Significantly lower BAL albumin levels were observed at 4 h and 12 h after acute bacterial challenge in the cessation group compared to switching and continued exposure groups (Fig. 5B). No differences between male and female mice were observed in the modulation of total BAL protein levels or albumin leak in any exposure conditions (Supplemental Figure E3A, B).

### Switching from cigarettes to HTP did not modulate MPO activity but augmented lung NE levels

**Fig. 6:**
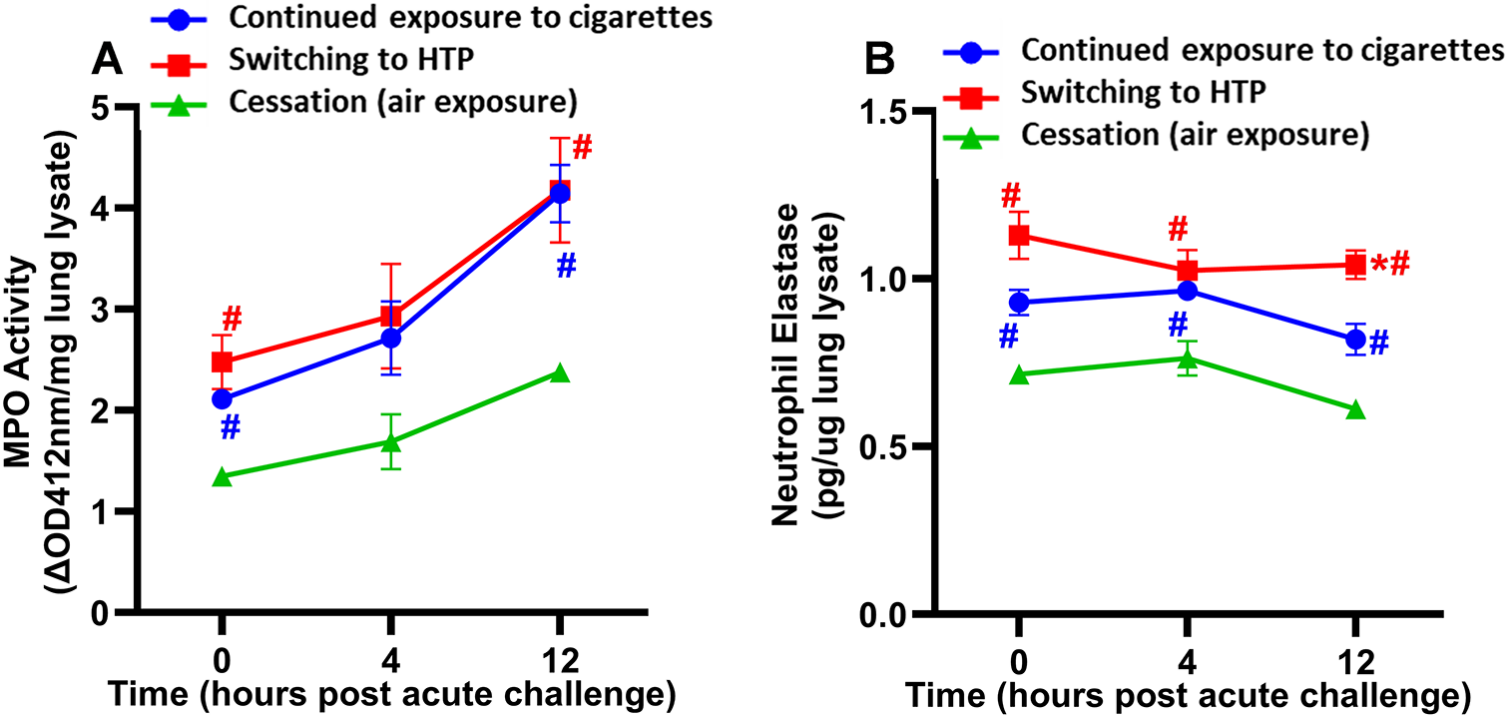
Measurement of MPO activity and NE levels in lung lysates prepared from the lungs of exposed-mice infected with NTHI for 0, 4, and 12 h. Switching from cigarettes to HTP did not modulate MPO activity whereas cessation was beneficial (**A**) and switching augmented lung NE levels (**B**). Difference between groups is considered significant at *p* < 0.05 using the Kruskal-Wallis test corrected for multiple comparisons by controlling the False Discovery Rate using Two-stage linear step-up procedure of Benjamini, Krieger and Yekuieli post-test. Desired FDR was adjusted at Q = 0.1. *Statistically significant difference from continued exposure to cigarettes (blue). # Statistically significant difference from cessation (green). Numbers of mice used for MPO assay: continued exposure to CS group (*n* = 8 mice at 0, *n* = 8 mice at 4 h and *n* = 7 mice at 12 h timepoint); for switching to HTP group (*n* = 8 mice at each 0, 4 and 12 h timepoints), and for cessation group (*n* = 8 mice at each 0, 4 and 12 h timepoints). Numbers of mice used for NE assay: continued exposure to CS group (*n* = 8 mice at 0, *n* = 10 mice at 4 h and *n* = 8 mice at 12 h timepoint); for switching to HTP group (*n* = 10 mice at each 0, 4 and 12 h timepoints), and for cessation group (*n* = 8 mice at 0, *n* = 10 mice at 4 h and *n* = 8 mice at 12 h timepoint). Results are depicted as mean ± SE

There were no significant differences in MPO activity between switching and continued exposure groups at any time point after acute bacterial challenge (Fig. 6A). MPO activity in lung tissue at 0 h and 12 h after acute bacterial challenge was significantly lower in the cessation group than in switching and continued exposure groups (Fig. 6A). No differences in the modulation of MPO activity were observed between female and male mice within any of the exposure groups (Supplemental Figure E4A).

We noted that the levels of NE at 12 h after acute bacterial challenge were significantly higher in the switching group than in the continued cigarette exposure group (Fig. 6B). The NE levels were noticeably higher (although non-significantly different) in female than in male mice (Supplemental Figure E4B). Further, the cessation group showed significantly lower levels of NE in lung tissue at all three time points after acute bacterial challenge compared to switching and continued cigarette exposure groups (Fig. 6B).

## Discussion

Due to the increased worldwide popularity of HTPs like IQOS and their marketing as a ‘safer’ alternative to combustible cigarettes owing to industry-generated safety data, we assessed for the first time whether switching from chronic cigarettes to HTPs can indeed reverse or minimize immunocompromising effects of cigarette smoke inhalation. Our study provides new evidence on how switching exposure from chronic inhalation of cigarette smoke to aerosols generated from HTP impacts the pulmonary inflammatory responses to an acute respiratory bacterial infection. Overall, we generally observed that detrimental pulmonary effects associated with chronic exposure to cigarette smoke were not considerably improved after switching to HTP. However, there was an overall marked improvement upon complete cessation of cigarette smoke exposure.

Our study’s major finding is that switching from combustible cigarettes to IQOS only moderately reduced the induction of lung endothelial damage compared to continued chronic cigarette smoke exposure. Indeed, switching from cigarettes to IQOS marginally increased the clearance of NTHI bacteria from the lungs of mice. Interestingly, this improvement in bacterial clearance in switching group correlated with neutrophil influx into the lung but did not correlate with markers of endothelial damage. Thus, the increased neutrophil numbers observed in the switching group may be beneficial in mitigating the bacterial infection. Other outcomes of respiratory infections that improved after switching from cigarettes to IQOS included inflammatory T-cell infiltration into the lungs, the content of total proteins accumulated in BAL, and albumin leak. However, all improvements upon switching were manifested only at later time points post bacterial challenge and were still suppressed at early times, as seen in animals continuously exposed to cigarette smoke.

It is important to note that several outcomes of pulmonary damage and inflammation induced by switching to IQOS were comparable to those induced by continued exposure to cigarette smoke. This is consistent with the results of our previous studies, which demonstrated that a proinflammatory pulmonary milieu induced by HTP in naïve animals was comparable to that induced by cigarettes [19, 22]. Importantly, switching from cigarettes to IQOS did not benefit lung epithelial-cell damage at all. For example, switching from cigarettes to IQOS did not improve lung myeloperoxidase and neutrophil elastase levels (markers for lung inflammation and damage). This could be because IQOS aerosols contain some harmful and potentially harmful toxic components as those contained in cigarette smoke that are responsible for inducing such a detrimental effect. In fact, there is evidence indicating that IQOS may not be a safe tobacco alternative as the manufacturer claims it to be. The presence of nicotine and other harmful chemicals in HTPs might be still sufficient to induce toxic respiratory effects like those induced by cigarette smoke [25–27].

Smoking cessation has been demonstrated to significantly overcome the immunosuppressive effects of cigarette smoke and reduce pulmonary bacterial load post-acute challenge [9, 28]. Since no reports have directly compared the pulmonary effects of switching to HTPs vs. complete cessation of cigarette smoking, our secondary aim was to address this critical knowledge gap and provide a nuanced perspective. We found that switching from cigarettes to IQOS was markedly less beneficial than the cessation of cigarette smoke exposure across multiple outcomes of respiratory infections measured in our study. Bacterial clearance, total lung immune-cell infiltration, inflammatory T-cell infiltration into the lungs, the content of total proteins in the BAL, and albumin leak were significantly compromised after acute bacterial challenge in the *switching* group compared to mice in the *cessation* group. We also found that lung endothelial damage after switching from cigarettes to IQOS did not match the efficiency by which cessation of cigarette exposure reduced it. At the cellular level, the possible mechanism of this detrimental outcome in mice that were switched to IQOS inhalation could be due to impaired phagocytosis, thereby leading to the persistence of NTHI bacteria in the lung as has been demonstrated in the case of cigarette smoke [9, 19, 29, 30]. Conversely, compared to the cessation of cigarette smoke exposure, exclusive IQOS inhalation or switching to IQOS can potentially enhance susceptibility to respiratory infections. It can augment the persistence of bacteria in the lungs post-infection, thereby leading to more lung inflammation and associated lung damage. Indeed, a study demonstrated that IQOS exposure increases susceptibility to respiratory infections by enhancing pneumococcal adhesion to nasal epithelial cells [31]. This study, however, was an in vitro investigation using primary nasal epithelial cells that were given acute exposure to IQOS extract. Our study has confirmed those findings in in vivo experimental conditions. Our findings corroborate reports revealing adverse pulmonary health effects following the use of heated tobacco products [32, 33].

Our study has several limitations when extrapolating results presented to real-life exposure in humans. The animal exposure methodology in the present investigation was based on a whole-body exposure system, and nose-only exposure may be more suitable to simulate real-life human exposure. Nevertheless, our routine sampling did not detect nicotine deposited on animal fur, thus indicating that inhalation was a primary route of exposure to animals. Since we selected a popular brand of HTP, it remains to be evaluated whether our findings can be generalized to other types and brands of alternative tobacco products. Finally, studies are needed to evaluate the effects of other alternative tobacco products (e.g., e-cigarettes) in switching settings and compare them to the effects of HTP switching. Further, since epidemiological studies have consistently shown high rates of concurrent use of multiple tobacco products [34–36], therefore the impact of concurrent exposures to two products (HTPs plus combustible cigarettes and HTPs plus e-cigarettes) on pulmonary health needs to be evaluated and compared to exclusive exposure to alternative tobacco product and abstinence from all tobacco products.

## Conclusions

While HTPs are being promoted as safe alternatives to combustible smoking and have become increasingly popular, the pulmonary health effects associated with their use when compared to smoking combustible tobacco cigarettes are unknown. The present study provides evidence on how switching from combustible cigarette smoking to HTPs impacts the outcomes of acute respiratory infection including pulmonary inflammatory responses, clearance of lung pathogen, lung endothelial and epithelial damage, and markers of lung inflammation and damage. The study demonstrates significantly compromised clearance of bacteria from the lungs post-acute challenge occurred in both the *switching* group and in mice continuously exposed to cigarette smoke. Our study has important implications for regulating HTP products and communicating their potential risks and benefits to users. In the US, the mandate for FDA approval of MRTP applications requires applicants to precisely demonstrate that their consumer products reduce harm and improve overall public health. While the primary purpose of IQOS marketing was to reduce tobacco harm associated with traditional cigarette smoking, our current findings highlight that switching to IQOS inhalation does not produce significant beneficial effects in improving cigarette smoke-induced detrimental effects in the lung. Further, our current findings corroborate conclusions drawn from previous studies done by us and others in the field [19, 21, 22, 32, 33] that indicate that IQOS is as detrimental to pulmonary health as are combustible cigarettes. Importantly, our study concludes that IQOS switching is not a valuable substitute for cessation.

## Electronic supplementary material

Below is the link to the electronic supplementary material.

Supplementary Material 1

Supplementary Material 2

## Data Availability

No datasets were generated or analysed during the current study.

## Abbreviations

HTP: Heated tobacco products
BAL: Broncho alveolar lavage
MPO: Myeloperoxidase
NE: Neutrophil elastase
NTHI: Nontypeable *Haemophilus influenzae*
