# Supplementary material for: Can switching from cigarettes to heated tobacco products reduce consequences of pulmonary infection?
Source: Respir Res. 2024 Oct 19;25:381. doi: 10.1186/s12931-024-02992-y (PMC11491011; doi:10.1186/s12931-024-02992-y)
Supplement: Supplementary file 1 — Supplementary Material 1 [file 12931_2024_2992_MOESM1_ESM.pptx]

## Slide 1
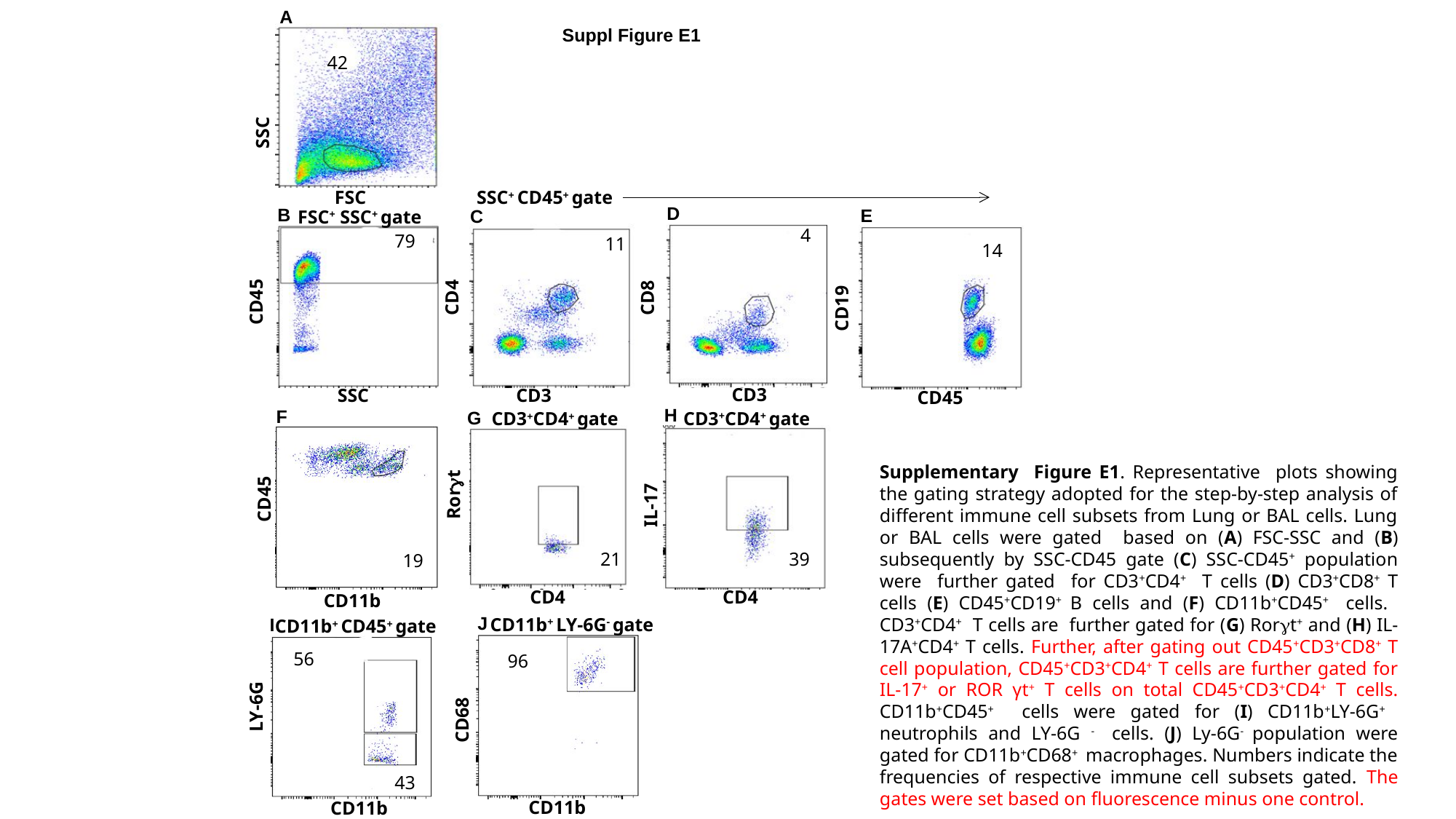

A
Suppl Figure E1
42
SSC
FSC
SSC+ CD45+ gate
D
B
E
C
FSC+ SSC+ gate
4
79
11
14
CD4
CD8
CD45
CD19
CD3
SSC
CD3
CD45
H
F
G
CD3+CD4+ gate
CD3+CD4+ gate
Supplementary Figure E1. Representative plots showing the gating strategy adopted for the step-by-step analysis of different immune cell subsets from Lung or BAL cells. Lung or BAL cells were gated based on (A) FSC-SSC and (B) subsequently by SSC-CD45 gate (C) SSC-CD45+ population were further gated for CD3+CD4+ T cells (D) CD3+CD8+ T cells (E) CD45+CD19+ B cells and (F) CD11b+CD45+ cells. CD3+CD4+ T cells are further gated for (G) Rorgt+ and (H) IL-17A+CD4+ T cells. Further, after gating out CD45+CD3+CD8+ T cell population, CD45+CD3+CD4+ T cells are further gated for IL-17+ or ROR γt+ T cells on total CD45+CD3+CD4+ T cells. CD11b+CD45+ cells were gated for (I) CD11b+LY-6G+ neutrophils and LY-6G - cells. (J) Ly-6G- population were gated for CD11b+CD68+ macrophages. Numbers indicate the frequencies of respective immune cell subsets gated. The gates were set based on fluorescence minus one control.
Rorgt
CD45
IL-17
21
39
19
CD4
CD4
CD11b
J
CD11b+ LY-6G- gate
I
CD11b+ CD45+ gate
56
96
LY-6G
CD68
43
CD11b
CD11b

## Slide 2
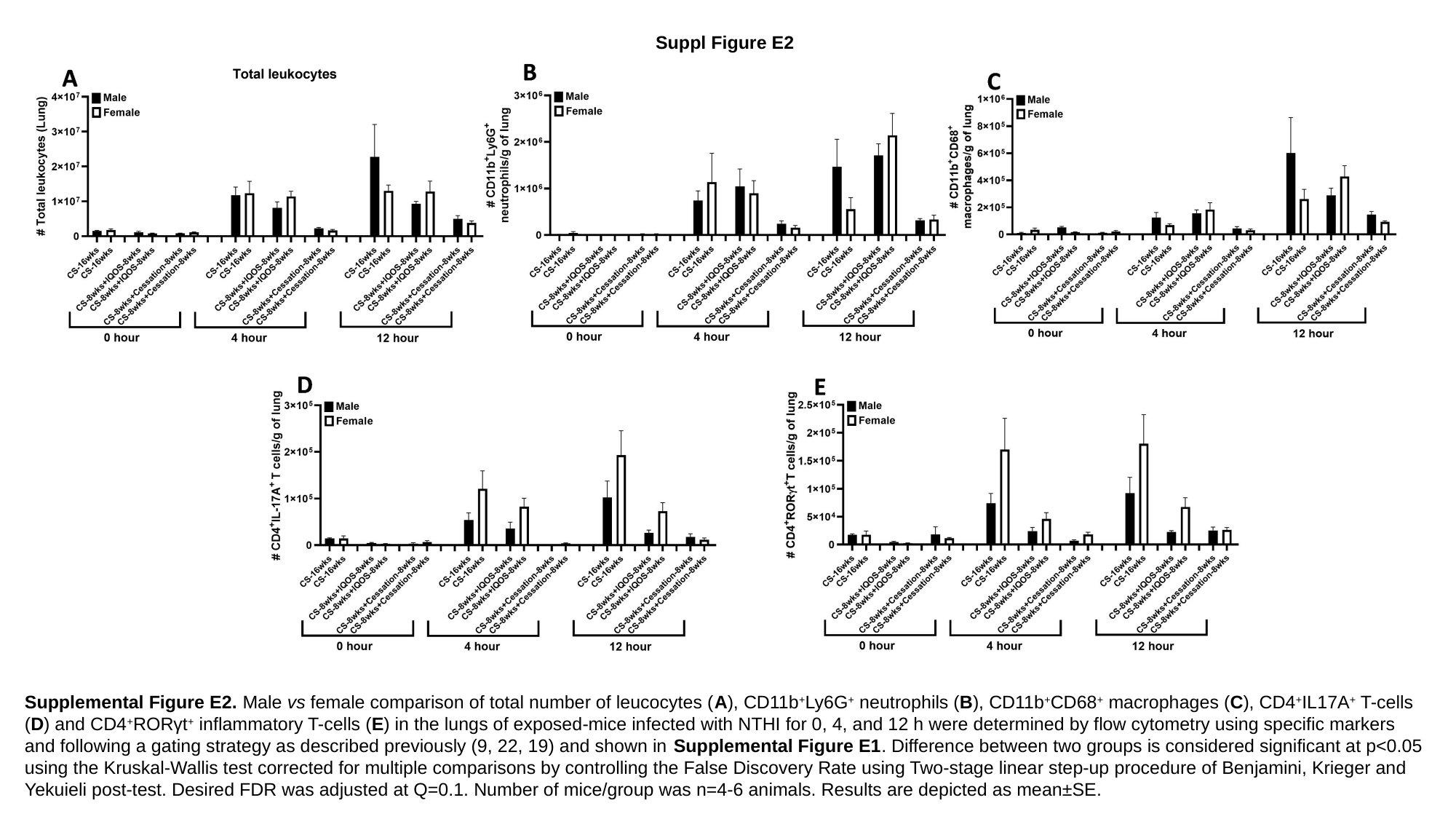

Suppl Figure E2
Supplemental Figure E2. Male vs female comparison of total number of leucocytes (A), CD11b+Ly6G+ neutrophils (B), CD11b+CD68+ macrophages (C), CD4+IL17A+ T-cells (D) and CD4+RORγt+ inflammatory T-cells (E) in the lungs of exposed-mice infected with NTHI for 0, 4, and 12 h were determined by flow cytometry using specific markers and following a gating strategy as described previously (9, 22, 19) and shown in Supplemental Figure E1. Difference between two groups is considered significant at p<0.05 using the Kruskal-Wallis test corrected for multiple comparisons by controlling the False Discovery Rate using Two-stage linear step-up procedure of Benjamini, Krieger and Yekuieli post-test. Desired FDR was adjusted at Q=0.1. Number of mice/group was n=4-6 animals. Results are depicted as mean±SE.

## Slide 3
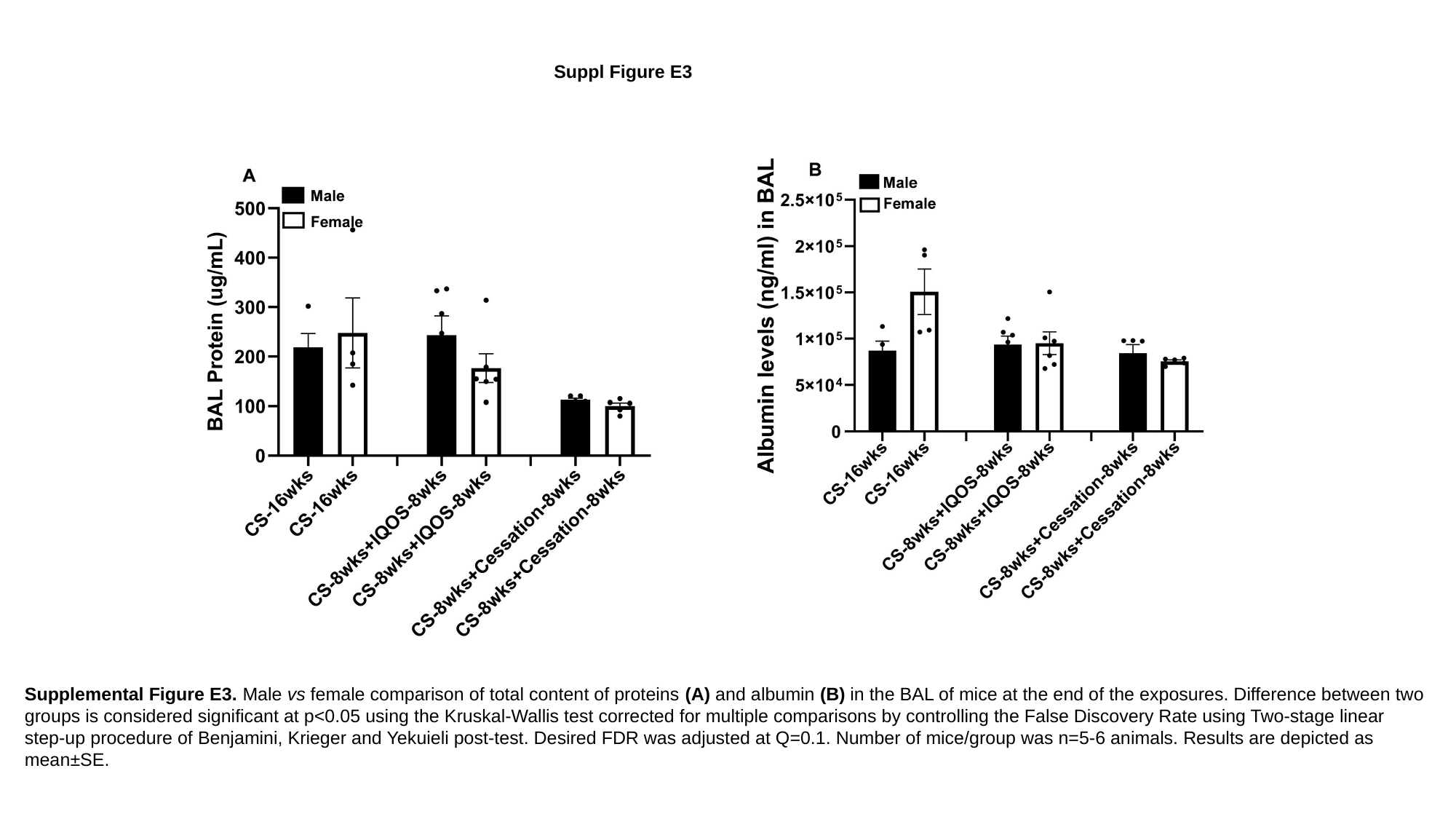

Suppl Figure E3
Supplemental Figure E3. Male vs female comparison of total content of proteins (A) and albumin (B) in the BAL of mice at the end of the exposures. Difference between two groups is considered significant at p<0.05 using the Kruskal-Wallis test corrected for multiple comparisons by controlling the False Discovery Rate using Two-stage linear step-up procedure of Benjamini, Krieger and Yekuieli post-test. Desired FDR was adjusted at Q=0.1. Number of mice/group was n=5-6 animals. Results are depicted as mean±SE.

## Slide 4
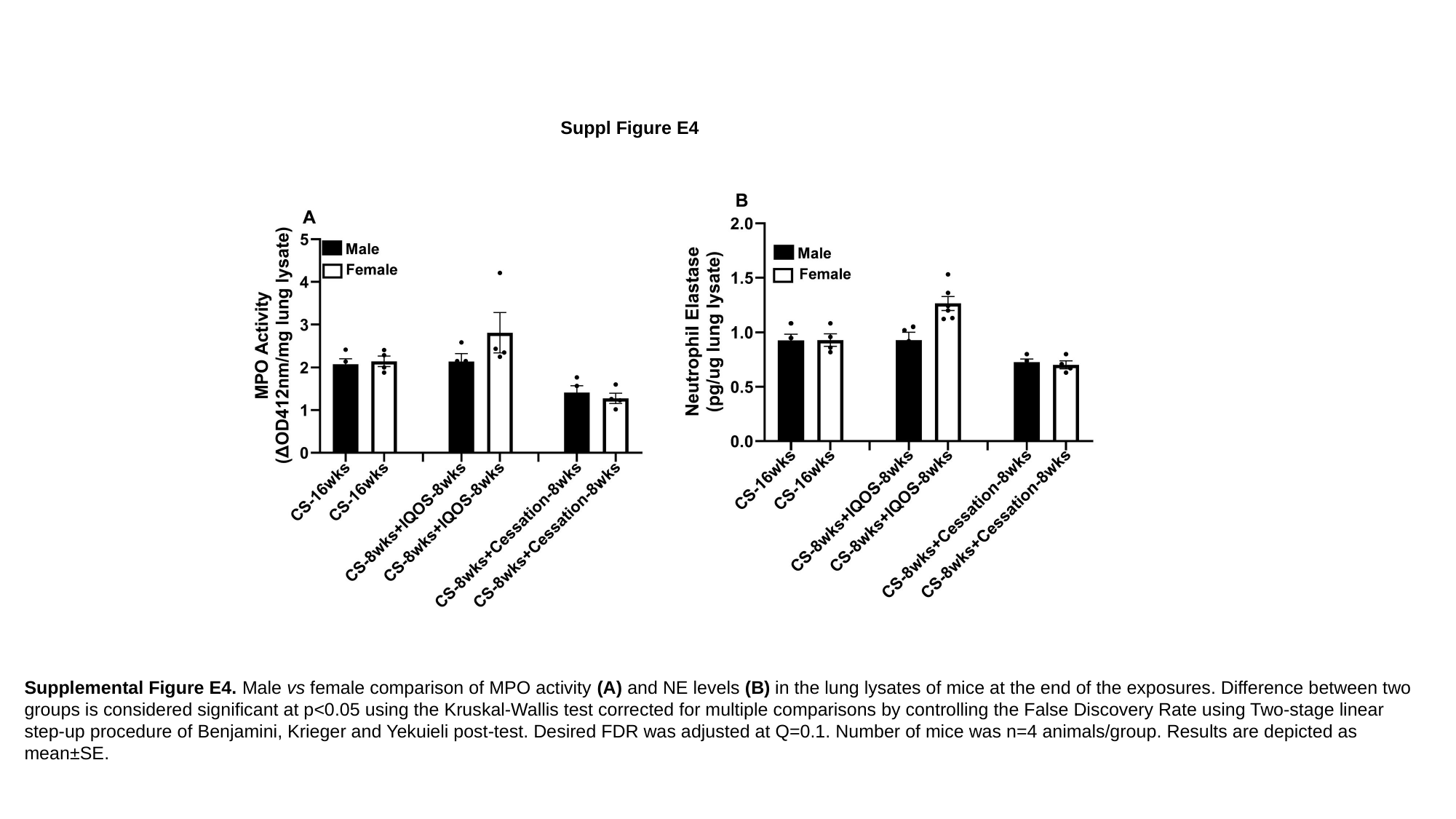

Suppl Figure E4
Supplemental Figure E4. Male vs female comparison of MPO activity (A) and NE levels (B) in the lung lysates of mice at the end of the exposures. Difference between two groups is considered significant at p<0.05 using the Kruskal-Wallis test corrected for multiple comparisons by controlling the False Discovery Rate using Two-stage linear step-up procedure of Benjamini, Krieger and Yekuieli post-test. Desired FDR was adjusted at Q=0.1. Number of mice was n=4 animals/group. Results are depicted as mean±SE.

## Slide 5
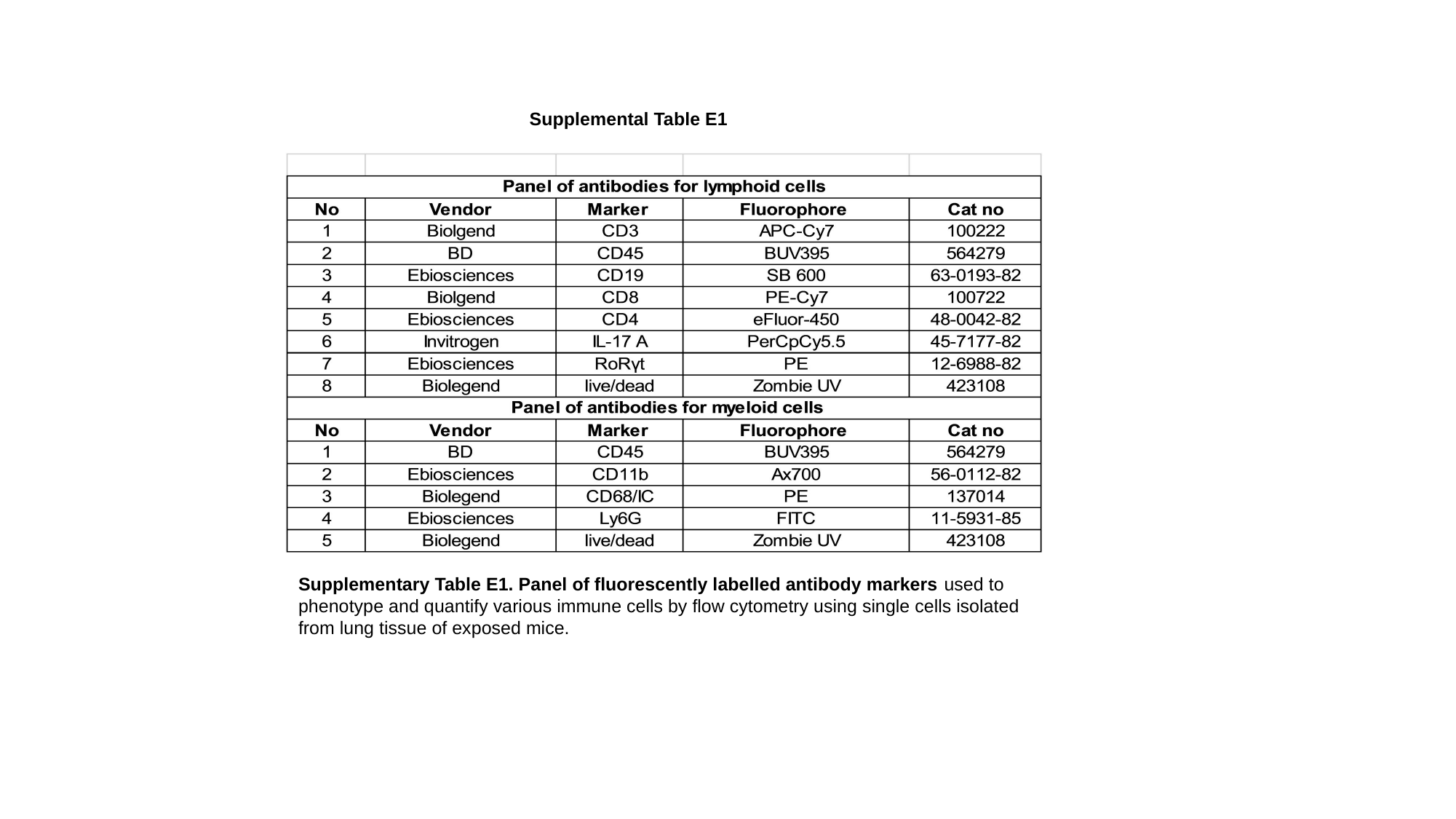

Supplemental Table E1
Supplementary Table E1. Panel of fluorescently labelled antibody markers used to phenotype and quantify various immune cells by flow cytometry using single cells isolated from lung tissue of exposed mice.
