# Supplementary material for: Can switching from cigarettes to heated tobacco products reduce consequences of pulmonary infection?
Source: Respir Res. 2024 Oct 19;25:381. doi: 10.1186/s12931-024-02992-y (PMC11491011; doi:10.1186/s12931-024-02992-y)
Supplement: Supplementary file 2 — Supplementary Material 2 [file 12931_2024_2992_MOESM2_ESM.docx]

**Supplementary Materials and Methods**

**Tobacco Products**

We selected the IQOS brand since this is the only HTP authorized by the US FDA. The product (version 2.4) was available in the US market during our study. To generate aerosols from the IQOS system, we used Marlboro Silver HEETS tobacco inserts. IQOS devices and Marlboro HEETS were purchased from Altria (Richmond, VA, USA) in April 2020. Combustible cigarettes were reference tobacco cigarettes 1R6F purchased from the University of Kentucky (Lexington, KY, USA). All 1R6F cigarettes and Marlboro inserts for HTP were conditioned at 22.0±1.0°C with a relative humidity of 60.0±2.0% for 48 hours before exposure experiments following the CORESTA 21 protocol [1].

**Animals**

Eight-week-old C57BL/6NCr mice were purchased from Charles River Laboratory (Wilmington, MA, USA) and housed under pathogen-free conditions at Roswell Park Comprehensive Cancer Center (Buffalo, NY, USA) with a light/dark cycle of 12/12 hours. Animal procedures were approved by the Institutional Animal Care and Use Committee and complied with all state, federal, and NIH regulations.

**Animal Exposure Conditions**

Eight-week old C57BL/6NCr mice (n=120; 60 males and 60 females) were pre-exposed for 8 weeks to cigarette smoke. After 8 weeks, mice were randomly assigned to three different exposure conditions for the next 8 weeks: 1) continued exposure to cigarette smoke, 2) cessation group (air exposure), or 3) product switching group (HTP), (n=40 mice/group; 20 male and 20 female). Animals were placed in exposure cages, each cage holding 10 males and 10 females in four quadrants (5 same-sex animals/quadrant). Mice were rotated clockwise daily to ensure uniform exposure.

To generate aerosols from HTP and cigarette smoke, we used an automatic JB2090 smoking machine (CH Technologies, Westwood, NJ, USA). Aerosols from each product were generated following the Health Canada Intense puffing regiment (puff of 55 mL volume and 2-sec duration taken every 30 secs [2] clustered as described in **Table 1**. Freshly generated aerosols were transferred to animal exposure cages using a tubing system and peristaltic pump. After aerosols from a cluster of puffs were delivered to animal exposure cages, filtered air was consistently supplied to the same cages. We used the same system for exposure to air (control) but did not connect any tobacco product.

Exposure conditions were strictly monitored during each experiment and included measurement of airborne total particulate matter (TPM), PM_5.0_, airborne nicotine, and nicotine deposited on cage surfaces and animal fur. TPM was constantly monitored during experiments using the CEL-712 Microdust Pro monitor (Casella, UK). The concentration of particulates (PM5.0) was determined gravimetrically using a 5.0mm GLA-5000 PVC membrane filter placed inside the 37mm PVC filter holder (SKC Inc., Eighty-Four, PA, USA). We also collected air samples inside exposure chambers to measure airborne nicotine concentration. Nicotine was sampled daily using an active sampling technique on XAD-4 sorbent tubes (SKC Inc.) with a flow rate of 1.7 L/min. Sorbent tubes were processed following NIOSH 2551 protocol, [3] and nicotine was analyzed using gas chromatography [4]. Samples of animal fur and wipes of the wall surface area inside the exposure chamber were also collected to measure the potential deposition of nicotine [4]. Temperature, atmospheric pressure, and humidity sensors placed inside the exposure chamber continuously monitored exposure conditions during experiments.

We decided to expose animals to an equivalent dose of nicotine delivered from all tested products. Nicotine equivalency was determined by quantifying serum cotinine levels (a primary nicotine metabolite) in blood samples collected 30 mins post-exposure, as described in detail previously [4 ]. Since aerosols emitted from the three tested products had different physicochemical properties, we performed a series of pilot experiments to calibrate the exposure system and develop a unique puffing protocol for each tested product (data not shown). Thus, despite differences in the puffing protocols used in our experiments, we achieved equivalent exposure to nicotine from all tested products (**Table 1**).

Importantly, higher levels of cotinine, a major nicotine metabolite, were observed in female mice compared to male mice in our experiments (**Table 1**). This is consistent with the sex-dependent pharmacokinetics of nicotine metabolism in mice, as several studies have shown a faster rate of nicotine elimination from the liver of female than male mice (5-7 ).

**Sample collection timeline**

Sixteen hours after exposures had ended, 20 randomly selected animals from each group were given intratracheal FITC-dextran (10ug/mouse in 50uL of 1x PBS) and levels of plasma FITC-dextran were measured 1 hour later by drawing blood samples from mice via the retro-orbital route. Sixty-five hours after exposures had ended, all animals in each exposure condition (n=40/group, 20 males and 20 females) (**Figure 1**) were given an acute intratracheal bacterial infection with NTHI (10^6^ cfus/mouse in 50uL 1x PBS) and euthanized 0, 4, and 12 hours later to harvest the BAL and lungs.

**Assessment of Pulmonary Inflammation**

The pulmonary inflammatory microenvironment was evaluated by quantifying immune-cell infiltration in the lungs. Isolated lung leukocytes were used in flow cytometry experiments to phenotype various immune cells using specific fluorochrome-conjugated antibodies (list provided in **Supplemental Table E1**) using the gating strategy given in **Supplemental Figure E1**.

**Isolation of leukocytes from the lung**

Leukocytes from mouse lungs were isolated, as reported previously [8, 9 ]. Briefly, lung tissue was minced in a 60 mm glass dish into small pieces by a sterile curved scissor. The resulting tissue slurry was mixed and incubated in 1x PBS solution containing 1 mg/mL Type IA-S collagenase and 50 U/mL DNase I (Sigma-Aldrich, St. Louis, MO, USA) and placed on a rotator incubator for one hour at 37°C. The resultant single-cell suspension was filtered through a 40μm cell strainer to remove debris/undigested tissue and centrifuged at 2,000 rpm for 5 minutes at room temperature. The cell pellet was resuspended in 9 mL complete RMPI-1640 media containing 10% FBS and then underlaid with 5 mL Ficoll-Paque (GE Healthcare, Chicago, IL, USA) and centrifuged with the brake off at 1,700 rpm for 20 minutes at room temperature. Leukocytes at the interface were collected, centrifuged as above, and washed in 1x PBS to remove residual Ficoll-Paque, counted, and stained with cell-specific antibodies as described below.

**Multicolor flow cytometry**

Lungs were harvested at euthanasia, and lung tissue was digested to isolate leukocytes for flow cytometry, as stated above and reported previously [4,8, 9]. Cells were stained with cell type-specific antibodies to determine the numbers and phenotype of various immune subsets by flow cytometry analysis. Briefly, 0.5 million cells/sample were stained with specific anti-mouse fluorochrome-tagged antibodies in 0.1 mL of FACS staining buffer (1% BSA in PBS) for 30 min at 4°C and subsequently washed in FACS buffer before fixing with Cytofix (BD Biosciences, CA, USA). For intracellular staining, cells were permeabilized in permeabilization buffer (BD Biosciences) and then stained with specific antibodies as described previously [4,8, 9 ]. After washing and fixing, all the samples were resuspended in 0.1 mL of FACS buffer and stored in the dark at 4^o^C until acquisition. Samples were acquired within 24 hours of staining using LSRII-A flow cytometer, and the data was analyzed using FlowJo software. The list and source of fluorochrome-conjugated antibodies are provided in **Supplemental Table E1**, and the gating strategy is shown in (**Supplemental Figure E1**).

**Estimation of lung damage**

Lung epithelial-cell integrity was measured by quantifying in the BAL the total amount of proteins and the levels of albumin built after systemic to bronchoalveolar space leak as described previously [4,8, 9]. Further, lung endothelial integrity was estimated by measuring the bronchoalveolar to systemic leak of intratracheally-instilled FITC-dextran as described previously [9, 10]. We measured the levels of NE and MPO in lung tissue using a procedure described previously [9] and mentioned below. Details of each of these methods are given below.

**FITC-dextran leak**

The systemic leak of FITC-dextran from bronchoalveolar space was quantified to evaluate lung endothelial damage as described previously [9, 10]. Fifty microliters of 200 mg/mL FITC-dextran in 1x PBS were instilled in each mouse *via* the intratracheal route (10 μg/mouse). An hour later, mice were anesthetized and bled retro-orbitally, and plasma was collected by centrifugation. FITC fluorescence in the plasma was determined using Exci485/Emi528 wavelengths in a Synergy H1 Hybrid plate Reader (Agilent, Santa Clara, CA, USA). A standard curve was generated using known concentrations of FITC-dextran in 1x phosphate buffer saline to calculate the FITC-dextran levels in a sample. Using a straight-line curve equation, the unknown sample concentration was determined using the slope and intercept measurements from the standard curve.

**Total BAL protein determination**

Total protein levels in the BAL were measured using a BCA protein assay per the manufacturer’s instructions (Cat#23225; ThermoFisher Scientific, Waltham, MA, USA). Known bovine serum albumin concentrations were used to prepare a standard curve to calculate unknown concentrations in a sample using a straight-line equation.

**Albumin leak measurement**

Albumin levels in the BAL (a surrogate marker of lung epithelial cell damage) were measured by albumin-ELISA using Bethyl Laboratories (Montgomery, TX, USA) reagents. Following the manufacturer’s instructions, plates were developed using a 3,3’,5,5’-tetramethylbenzidine (TMB) solution from eBioscience Inc. (San Diego, CA, USA), and the absorbance was read at 450 nm in a Synergy H1 Hybrid plate Reader as described previously [9, 10].

**Myeloperoxidase (MPO) assay**

To measure MPO activity, we used an MPO activity assay kit from Abcam (Cat No. #ab105136; Cambridge, EN, UK) as we described previously [9, 10]. In this assay, MPO in a sample generates HClO (hypochlorous acid) from H_2_O_2_ and Cl^-^ ion that produces taurine chloramine after reacting with taurine. Taurine chloramine reacts with chromophore TNB and eliminates the color at 412 nm. Thus, the absorbance measured at 412 nm in a sample is inversely proportional to the amount of MPO present. Briefly, a sample reaction was initiated either in the presence or absence of MPO substrate in a 96-well plate and incubated at 25^o^C for 1 hour to generate taurine chloramine, after which the reaction was stopped. Samples were incubated with TNB reagent for 10 minutes at room temperature following the kit’s instructions and as described previously [9, 10]. Absorbance was measured at 412 nm in a Synergy H1 Hybrid plate Reader, and the data were depicted as the difference in the absorbance in a sample reaction carried either in the presence or absence of substrate reagent. The change in absorbance (final data) at 412 nm (∆OD_412nm_ = (OD_412nm_) sample blank (absence of substrate) minus (OD_412nm_) sample (presence of substrate). OD- optical density.

**Neutrophil elastase (NE) assay**

Neutrophil elastase (NE) levels in the lung tissue were measured by Neutrophil Elastase/ELA2 DuoSet ELISA kit (Cat. No. #DY4517-05; R&D Systems, Minneapolis, MN, USA) following the manufacturer’s kit protocol and as described previously [9, 10]. ELISA plates were coated with anti-NE Ab and incubated overnight at room temperature. The next day, plates were blocked and washed, and the samples or standards diluted in 1x PBS were added to the plate and incubated for 2 hours at room temperature. After washing 3 times, plates were incubated with detection Ab at room temperature for 2 hours, followed by washing the plates 3 times, adding streptavidin-HRP solution, and incubating the plate in the dark for 20 minutes at room temperature. Plates were then washed 5 times, a substrate solution (mixture of H2O2 and tetramethylbenzidine) was added to each well, and the plate was incubated in the dark for 20 minutes at room temperature. Stop solution (2N H_2_SO_4_) was added to each well, and the absorbance of the plate was measured at 450 nm in a Synergy H1 Hybrid plate Reader. Optical imperfections in the plate were corrected by reading at 540 or 570 nm and subtracting these from 450 nm readings as per kit recommendations.

**Acute pulmonary NTHI challenge**

Acute lung infections were done using a freshly grown NTHI strain 1479, a clinical isolate from a COPD exacerbation as described previously [8, 9]. Each mouse received a single intratracheal instillation of NTHI (1 x 106 live bacteria in 1x PBS) in a final volume of 50 μL, and animals were euthanized 0, 4, or 12 h later to harvest the BAL and lungs for evaluating markers associated with acute inflammation and lung damage.

**NTHI clearance**

Bacterial clearance in the lungs was quantified as described previously [8, 9]. Mice were euthanized 0, 4, or 12 hours post-acute NTHI challenge to harvest lungs under sterile conditions. Lung homogenates were prepared by gentle homogenization in 1 mL PBS on ice. Serial dilutions of lung homogenates were plated onto chocolate agar plates and incubated at 35°C, 5% CO_2_ for 16 hours. NTHI bacterial colonies were counted, and %clearance rates were calculated using the formula %NTHI clearance= {[100 ̶ [((no. of bacterial colonies) x (dilution factor) x (total volume (μL) of lung homogenate)) / (volume (μL) of lung homogenate used for plating)]}%.

**Supplemental References**

1. ISO 3402:1999. Tobacco and Tobacco Products – Atmosphere for Conditioning and Testing. Geneva, Switzerland: International Organization for Standardization (ISO).
2. ISO/TC 126, Tobacco and tobacco products. ISO and Health Canada intense smoking parameters - Part 2: Examination of factors contributing to variability in the routine measurement of TPM, water and NFDPM smoke yields of cigarettes. [ISO/TR 19478-2:2015(en), ISO and Health Canada intense smoking parameters — Part 2: Examination of factors contributing to variability in the routine measurement of TPM, water and NFDPM smoke yields of cigarettes](https://www.iso.org/obp/ui/#iso:std:iso:tr:19478:-2:ed-1:v1:en)
3. Nicotine: Method 2551, Issue 1, dated 15 January 1998. In: Ashley K and O’Connor PF, eds. NIOSH Manual of Analytical Methods (NMAM), 4th ed. Atlanta, GA: Centers for Disease Control and Prevention (CDC); 1994.
4. Bhat TA, Kalathil SG, Leigh N, Muthumalage T, Rahman I, Goniewicz ML, Thanavala YM. Acute Effects of Heated Tobacco Product (IQOS) Aerosol Inhalation on Lung Tissue Damage and Inflammatory Changes in the Lungs. Nicotine Tob Res 2021;23(7):1160-1167.
5. Arecco N, Clarke CJ, Jones FK, et al. Elastase levels and activity are increased in dystrophic muscle and impair myoblast cell survival, proliferation and differentiation. Sci Rep 2016;6:24708.
6. Czégény Z, Nagy G, Babinszki B, et al. Cbd, a precursor of THC in e-cigarettes. Sci Rep 2021;11:8951.
7. Lindén A, Laan M, Anderson GP. Neutrophils, interleukin-17A and lung disease. Eur Respir J 2005;25:159–72.
8. Bhat TA, Kalathil SG, Bogner PN, Miller A, Lehmann PV, Thatcher TH, et al. Secondhand smoke induces inflammation and impairs immunity to respiratory infections. J Immunol 2018;200(8):2927–2940.
9. Bhat TA, Kalathil SG, Leigh N, Hutson A, Goniewicz ML, Thanavala YM. Do alternative tobacco products induce less adverse respiratory risk than cigarettes? Respir Res 2023;24(1):261.
10. Bhat TA, Kalathil SG, Goniewicz ML, Hutson A, Thanavala Y. Not all vaping is the same: differential pulmonary effects of vaping cannabidiol versus nicotine. Thorax 2023;78(9):922-932.
